# Similarities and differences among Internet gaming disorder, gambling disorder and alcohol use disorder: A focus on impulsivity and compulsivity

**DOI:** 10.1556/JBA.3.2014.4.6

**Published:** 2014-12-18

**Authors:** SAM-WOOK CHOI, HYUN SOO KIM, GA-YOUNG KIM, YEONGJU JEON, SU MI PARK, JUN-YOUNG LEE, HEE YEON JUNG, BO KYOUNG SOHN, JUNG-SEOK CHOI, DAI-JIN KIM

**Affiliations:** ^1^Department of Psychiatry, Gangnam Eulji Hospital, Eulji University, Seoul, South Korea; ^2^Eulji Addiction Institute, Eulji University, Seoul, South Korea; ^3^Department of Psychiatry, Myongji Hospital, Goyang, South Korea; ^4^Clinical Research Center for Depression, Department of Psychiatry, St. Mary’s Hospital, the Catholic University of Korea, Seoul, South Korea; ^5^Department of Psychiatry, SMG-SNU Boramae Medical Center, Seoul, South Korea; ^6^Department of Psychiatry, Seoul National University College of Medicine, Seoul, South Korea; ^7^Department of Psychiatry, Seoul St. Mary’s Hospital, College of Medicine, the Catholic University of Korea, Seoul, South Korea

**Keywords:** behavioral addiction, Internet gaming disorder, gambling disorder, alcohol use disorder, alcohol use disorder, impulsivity, compulsivity

## Abstract

*Background and aims:* The aim of the present study was to test the impulsivities and compulsivities of behavioral addictions, including Internet gaming disorder (IGD) and gambling disorder (GD), by directly comparing them with alcohol use disorder (AUD) and a healthy control (HC) group. *Methods:* We enrolled male patients who were diagnosed with IGD, GD or AUD, with 15 patients per group, as well as 15 HCs. Trait impulsivity was measured using the Barratt Impulsiveness Scale version 11 (BIS-11). The stop-signal test (SST) from the Cambridge Neuro-psychological Test Automated Battery (CANTAB) was used to assess the patients’ abilities to inhibit prepotent responses. Compulsivity was measured using the intra–extra dimensional set shift (IED) test from the CANTAB. The Trail Making Test (TMT) was also used in this study. *Results:* The IGD and AUD groups scored significantly higher on the BIS-11 as a whole than did the HC group (*p* = 0.001 and *p* = 0.001, respectively). The IGD and AUD groups also scored significantly higher on the BIS-11 as a whole than did the GD group (*p* = 0.006 and *p* = 0.001, respectively). In addition, the GD group made significantly more errors (*p* = 0.017 and *p* = 0.022, respectively) and more individuals failed to achieve criterion on the IED test compared with the IGD and HC groups (*p* = 0.018 and *p* = 0.017, respectively). *Discussion:* These findings may aid in the understanding of not only the differences in categorical aspects between individuals with IGD and GD but also in impulsivity–compulsivity dimensional domains. *Conclusion:* Additional studies are needed to elucidate the neurocognitive characteristics of behavioral addictive disorders in terms of impulsivity and compulsivity.

## INTRODUCTION

Pathological gambling has been included in the DSM-IV in the past under the classification of “impulse-control disorders not elsewhere classified”, and it was included in the DSM-5 under a new name and classified under “gambling disorder (GD)” and “substance-related and addictive disorders”. Several studies have suggested that GD shares many similarities with substance addictions, including those involving natural histories, clinical features, comorbidities, neurobiological mechanisms, and treatment responses (Grant, Potenza, Weinstein & Gorelick, 2010). This is partially due to the fact that GD shares a number of common characteristics with substance addiction, including the poor performances of affected individuals on neurocognitive tasks, particularly with respect to impulsive choices, response tendencies and compulsive features (e.g., response perseveration and action with diminished relationship to goals or reward) (Grant et al., 2010; Leeman & Potenza, 2012).

In individuals with substance addiction and GD, impulsivity is at the root of a tendency to pursue short-term rewards (drugs or gambling) and it is a powerful mechanism in the early stages of addiction (Fernandez-Serrano, Perales, Moreno-Lopez, Perez-Garcia & Verdejo-Garcia, 2012; Leeman & Potenza, 2012; Verdejo-Garcia, Lawrence & Clark, 2008). If regular drug use or gambling behaviors occur over an extended period of time, reward-based learning mechanisms develop into compulsive behaviors (Leeman & Potenza, 2012).

Internet gaming disorder (IGD) also can be defined as a type of behavioral addiction (Cho et al., 2014; Demetrovics et al., 2012; Grant et al., 2010; Petry & O’Brien, 2013). IGD has been included in Section III of the DSM-5 with a list of proposed diagnostic criteria to encourage future research (Cho et al., 2014; Ko et al., 2014; Petry & O’Brien, 2013). This disorder shares core clinical features with GD, which include the continued engagement in the addictive behavior despite adverse consequences, the loss of control over the engagement in the behavior, and the presence of a craving state prior to engagement despite a lack of substance use (Muller, Beutel, Egloff & Wolfling, 2014). IGD also has been associated with diminished impulse control (Cao, Su, Liu & Gao, 2007; Choi et al., 2014; Ding et al., 2014; Yau, Potenza & White, 2013) with similar features to GD (Blanco et al., 2009; Chambers & Potenza, 2003; Kraplin et al., 2014; Steel & Blaszczynski, 1998).

To date, few studies exist describing the compulsivity of IGD (Dong, Lin, Zhou & Lu, 2014), although many studies have been performed assessing impulsivity and com-pulsivity in GD and substance addiction. Furthermore, no studies have been conducted on impulsivity and compul-sivity in IGD involving direct comparisons with GD and substance addiction.

The purpose of this study is to compare IGD, GD and alcohol use disorder (AUD) in terms of impulsivity and compulsivity. The examination of the similarities and differences of these addictive disorders will aid in the clarification of the psychopathological features of each behavior, such as IGD or GD, and help to identify specific target symptoms for the determination of more effective individual treatments.

## METHODS

### Participants and procedures

We enrolled patients who were diagnosed with IGD, GD or AUD, with 15 patients per group, as well as 15 healthy controls (HC). These patients all sought treatment at our clinics and complained of excessive Internet game use, problematic gambling behaviors, or alcohol-related problems. We restricted enrollment to male patients and healthy controls because males have a higher prevalence of problematic online game use compared with females (Ko, Yen, Chen, Chen & Yen, 2005). Patients were recruited from the outpatient clinic of the Seoul Metropolitan Government-Seoul National University (SMG-SNU) Boramae Medical Center and Gangnam Eulji Hospital at Eulji University in Seoul, South Korea.

Patients with IGD were diagnosed according to the DSM-5 criteria and were also assessed using the standardized Korean version of Young’s Internet Addiction Test (IAT) (Kim et al., 2003). We recruited patients with IAT scores of at least 70 who spent over 4 hours per day and 30 hours per week using Internet games. Although previous studies have associated excessive Internet use with an IAT score of at least 50 (Young, 1996), our inclusion criterion was made stricter to ensure that our sampled population had severe Internet addictions rather than being at high risk for this type of addiction. Young‘s IAT is in accordance with the DSM-IV criteria for pathological gambling and is widely used by investigators worldwide. Test items are rated on a 5-point scale, in which 1 indicates “very rarely” and 5 indicates “very frequently”. Total scores were calculated according to Young‘s method, and possible scores for all 20 items ranged from 20 to 100. All of the patients with IGD used the Internet primarily for online gaming.

GD patients who expressed interest in receiving treatment for gambling problems and who fulfilled the DSM-5 criteria for GD were recruited. The diagnoses were determined by a board-certified psychiatrist using a semi-structured interview process. The severity of GD was assessed using the Problem Gambling Severity Index (PGSI) from the Canadian Problem Gambling Index, which is a nine-item self-reporting assessment that has been demonstrated to be useful in both clinical and non-clinical settings (Loo, Oei & Raylu, 2011; Young & Wohl, 2011).

AUD was diagnosed using the Structured Clinical Interview for DSM-IV Disorders (SCID). The Alcohol Use Disorder Identification Test (AUDIT) was used to assess the severity of AUD (Sung, Lee, Song & Kim, 2011).

HC were recruited from the local community and had no history of psychiatric disorders. Subjects were enrolled in the GD, AUD or HC groups if they used Internet games for less than 2 hours per day. All of the patients were drug naïve.

To evaluate depression and anxiety symptoms, all participants were asked to complete the Beck Depression Inventory (BDI) (Beck, Ward, Mendelson, Mock & Erbaugh, 1961) and Beck Anxiety Inventory (BAI) (Beck, Epstein, Brown & Steer, 1988).

### Evaluation of impulsivity and compulsivity

Trait impulsivity was measured using the Korean version of the Barratt Impulsiveness Scale version 11 (BIS-11) (Patton, Stanford & Barratt, 1995). The BIS-11 consists of three factors: cognitive impulsiveness (making quick decisions), motor impulsiveness (acting without thinking), and non-planning impulsiveness (a lack of “futuring” or foresight) (Patton et al., 1995). Researchers have accepted the validity of this measurement.

Neurocognitive tests were also used to measure impul-sivity. The stop-signal test from the Cambridge Neuropsy-chological Test Automated Battery (CANTAB) (Robbins et al., 1998), which is a classic, computerized, stop-signal response-inhibition test that is used to assess an individual’s ability to inhibit a prepotent response, was used in this study.

Compulsivity was also measured using the intra–extra dimensional set shift test from the CANTAB, a test of rule acquisition and a reversal that was used to assess visual discrimination, attentional set-formation maintenance, and the assessment of the ability of the individual to shift and flexibly allocate attention (see http://www.cambridgecognition.com). The Trail Making Test (Seo et al., 2006), which is a traditional psychological test that assesses motor planning (type A) and cognitive inflexibility in relation to compul-sivity (type B), was also used in this study.

### Statistical analysis

To compare demographic variables among groups, one-way ANOVA was used, and a post-hoc analysis was performed using the Fisher’s least significant difference (LSD) test. One-way ANCOVA was conducted to examine differences among the groups with respect to impulsivity and com-pulsivity. Age was computed as a covariate. The IGD group was younger than the AUD and GD groups and the IGD group also had less years of education than the GD and HC groups. However, there was a significantly positive correlation between age and years of education (*r* = 0.511, *p* < 0.001). Thus, to avoid multicollinearity, we used ANCOVAs with only age as covariates for further analyses. All of the statistical analyses were carried out with SPSS version 19.0, and two-tailed tests were also used.

### Ethics

The institutional review boards of the SMG-SNU Boramae Medical Center and Eulji University approved the study protocol, and all subjects provided written informed consent before participation.

## RESULTS

### Demographic and clinical characteristics of the addicted and control groups

To compare the demographic variables among the groups, one-way ANOVA was used. Table 1 presents the descriptive statistics regarding the demographic and clinical characteristics of the studied groups. In the four groups, the one-way ANOVA showed statistically significant differences for age [*F*(3,56) = 7.114, *p* < 0.001] and education [*F*(3,56) = 4.874, *p* < 0.05]. Significant differences were also found for onset age [*F*(2,42) = 52.752, *p* < 0.001] and duration of illness [*F*(2,39) = 20.587, *p* < 0.001]. Unsurprisingly, the IGD group scored higher on the IAT and spent more weekday and weekend time on the Internet than did the HC group. Also as expected, the AUD group scored higher on the AUDIT-K than the other addiction and HC groups. The AUD group was also characterized by higher levels of anxiety as measured by the BAI. The PGSI mean is presented in Table 1.

**Table 1.** Demographic and clinical characteristics of the four subject groups

	IGD^a^ *(N=* 15)		GD^b^ *(N=* 15)		AUD^C^ *(N=* 15)		HC^d^ *(N=* 15)		*F(df)*	*p*	Significant group differences	***n^2^***
	Mean	*SD*	Mean	*SD*	Mean	*SD*	Mean	*SD*				
Age (years)	20.80 (16-36)	5.09	27.53 (17–34)	5.21	29.60 (20–39)	6.23	25.33 (15-33)	5.30	7.11	<.001	c > a b > a	.276
Education (years)	11.73 (9-16)	2.52	14.73 (9–18)	2.63	13.80 (9–18)	2.54	14.53 (9-16)	1.85	4.87	.004	d > a b > a	.207
Age of disease onset (years)	9.60 (5-15)	3.00	25.33 (17–33)	4.48	18.40 (10–30)	4.90	–	–	52.75	<.001	c > a b > a b > c	.715
Duration of illness (years)	11.20 (6-21)	3.69	3.13 (1–7)	1.64	10.58 (2–20)	5.47	–	–	20.59	<.001	a > b c > b	.514
IAT	75.40 (71-87)	4.39	–	–	23.25 (13–38)	6.22	24.67 (10–49)	11.94	184.43	<.001	a>d a> c	.904
Time for Internet use at weekday (hr/d)	5.13 (3-10)	2.20	–	–	1.21 (.5–2)	0.50	1.47 (0–3)	0.74	34.50	<.001	a > d a > c	.639
Time for Internet use at weekend day (hr/d)	7.07 (4-11)	2.09	–	–	1.33 (1–2)	0.49	1.53 (0–3)	.74	83.40	<.001	a > d a > c	.810
PGSI	–	–	19.87 (12–26)	3.83	–	–	–	–	–	–		
AUDIT	3.80 (0-20)	5.80	7.13 (0–21)	8.18	25.53 (0–21)	7.76	5.53 (0–14)	4.63	33.68	<.001	c > d c > a c > b	.643
BDI	18.07 (4-35)	9.87	13.40 (3–27)	8.74	27.13 (3–27)	16.13	3.47 (0–14)	3.56	13.03	<.001	a > d c > d c > b	.411
BAI	11.47 (0-28)	8.38	9.67 (1–28)	9.69	26.93 (1–28)	17.14	9.07 (0–35)	9.25	7.97	<.001	c > d c > a c > b	.299
TMT_A	22.47 (11-42)	7.90	24.67 (11–54)	11.27	28.20 (11–54)	26.02	18.93 (12–29)	4.62	0.707(3)	.552	No differences	

IGD: Internet Gaming Disorder; GD: Gambling Disorder; AUD: Alcohol Use Disorder; HC: Healthy Control; IAT: Young’s Internet Addiction Test; PGSI: Problem Gambling Severity Index; AUDIT: Alcohol Use Disorder Identification Test; BDI: Beck Depression Inventory; BAI: Beck Anxiety Inventory; TMT_A: Trails Making Test type A.

^a^ Internet Gaming Disorder; Gambling Disorder; ^c^ Alcohol Use Disorder; ^d^ Healthy Control

### Impulsivities of studied groups

One-way ANCOVA was conducted to examine the differences among the groups’ personality profiles, as measured by the BIS-11. Table 2 presents the differences in im-pulsivity among the groups (covariate included age). The results indicated the presence of significant differences on the BIS-11-cognitive, BIS-11-motor and BIS-11-non-planning scales in addition to the BIS-11 as a whole [main effect *F*(3,52) = 10.565, *p* < 0.001, covariate effect *F*(1,x) = 7.104, *p* = 0.010; main effect *F*(3,52) = 11.523, *p* < 0.001; covariate effect *F*(1,52) = 4.452, *p* = 0.040; main effect *F*(3,52) = 19.393, *p* < 0.001; covariate effect *p* = n.s; and main effect *F*(3,52) = 10.071, *p* < 0.001; covariate effect *F*(1,52) = 6.244, *p* = 0.016, respectively].

**Table 2.** Group differences for impulsivity

	IGD^a^ *(N* = 15)		GD^b^ *(N=l5)*		AUD^c^^(^*N*= 5)		HC^d^*(N* = 15)		*F(df)*	*P*	Significant group differences	*η**^2^***
	Mean	*SD*	Mean	*SD*	Mean	*SD*	Mean	*SD*				
BIS_11												
Total	72.93	9.61	55.13	14.90	74.15	15.40	56.93	9.66	10.071(3)	<.001	a > d c > d a > b c > b	.363
Cognitive	20.13	3.29	23.53	6.75	20.00	3.89	15.53	2.56	10.565(3)	<.001	a > d c > d b > d b > a	.374
Motor	24.07	4.40	16.53	3.80	25.31	7.36	17.47	4.67	11.523(3)	<.001	a > d c > d a > b c > b	.395
Non-planning	28.73	4.30	15.07	5.64	28.85	6.11	23.93	5.86	19.393(3)	<.001	c > d d > b a > b c > b	.523
SST												
DE stop and go	1.47	2.00	0.40	0.83	3.53	6.40	2.20	3.99	2.392(3)	.079	No differences	
DE stop sign	0.73	0.88	0.07	0.26	0.80	1.26	0.60	0.83	1.963(3)	.130	No differences	
DE go sign	2.00	2.36	0.33	0.62	2.73	5.24	1.60	3.50	1.571(3)	.207	No differences	
Successful stop ratio	0.52	0.07	0.58	0.11	0.51	0.12	0.62	0.15	3.162(3)	.032	d > a d > c	.147
Successful stop subblock	0.50	0.27	0.58	0.31	0.55	0.22	0.62	0.36	0.286(3)	.835	No differences	
Reaction time	503.09	208.89	567.86	173.74	495.67	207.54	566.66	271.05	0.471(3)	.704	No differences	
Stop signal Response Time half	175.02	81.07	151.75	40.66	202.32	130.79	171.92	51.69	1.048(3)	.379	No differences	

IGD: Internet Gaming Disorder; GD: Gambling Disorder; AUD: Alcohol Use Disorder; HC: Healthy Control; BIS_11: Barratt Impulsiveness Scale version 11; SST: Stop-Signal Test; DE: Direction Errors.

^a^ Internet Gaming Disorder; ^b^ Gambling Disorder; ^c^ Alcohol Use Disorder; ^d^ Healthy Control

Post-hoc LSD tests revealed that the IGD and AUD groups had significantly higher scores on the BIS-11 as a whole than did the HC group (*p* = 0.009 and *p* < 0.001, respectively). The IGD and AUD groups also had significantly higher scores on the BIS-11 as a whole than did the GD group (*p* = 0.011 and *p* < 0.001, respectively).

Post-hoc LSD tests also revealed that the IGD, GD and AUD groups had significantly higher scores on the BIS-11-cognitive compared with the HC group (*p* = 0.044, *p* < 0.001 and *p* = 0.001, respectively). The GD group scored significantly higher on the BIS-11-cognitive than did the IGD group (*p* = 0.003). Further post-hoc LSD tests revealed that the IGD and AUD groups had significantly higher scores on the BIS-11-motor compared with the HC (*p* = 0.007 and *p* < 0.001, respectively) and GD (*p* = 0.006 and *p* < 0.001, respectively) groups. These tests also revealed that the AUD group had significantly higher score on the BIS-11-non-planning compared with the HC group (*p* = 0.009). Interestingly, the IGD, AUD and HC groups had significantly higher scores on the BIS-11-non-planning than did the GD group (*p* < 0.001, *p* < 0.001 and *p* < 0.001, respectively). The ANCOVA results indicated significant differences among the proportions of successful stops on the stop-signal test [main effect *F*(3,55) = 3.162, *p* = 0.032]. Both the IGD and AUD groups showed decreased proportions of successful stops on the the stop-signal test compared with the HC group (*p* = 0.048 and *p* = 0.008, respectively). Covariate effects on all the stop-signal test variables were not significant.

### Compulsivities of studied groups

Table 3 presents the differences in compulsivity among the groups (covariate included age). The ANCOVA results indicated significant differences among the groups in the intra–extra dimensional set shift total error and the intra–ex-tra dimensional set shift total trial [main effect *F*(3,55) = 3.138, *p* = 0.033; main effect *F*(3,55) = 3.469, *p* = 0.022, respectively] values. Covariate effects on all the intra–extra dimensional set shift variables were not significant. Post-hoc LSD tests revealed that the GD group made significantly more errors (*p* = 0.015, *p* = 0.037 and *p* = 0.010, respectively) and that more individuals failed to achieve criterion on the intra–extra dimensional set shift compared with the IGD, AUD and HC groups (*p* = 0.012, *p* = 0.021 and p = 0.008, respectively). However, there were no significant differences among the groups on the Trail Making Test, particularly the Trail Making Test type B. In addition, covariate effects on all the Trail Making Test variables were not significant.

**Table 3.** Group differences for compulsivity

	IGD^a^ (Internet Gaming Disorder) *(N =* 15)		GD^b^ (Gambling Disorder) *(N =* 15)		AUD^C^ (Alcohol Use Disorder *N =* 15)		HC^d^ (Healthy Control) *(N =* 15)		*F(df)* group differences	*p*	significant	^η^2
	Mean	*SD*	Mean	*SD*	Mean	*SD*	Mean	*SD*				
IED												
Total error	14.13	9.07	27.93	20.13	17.47	12.35	14.67	8.16	3.138(3)	.033	b > a b > c b > d	.146
Total trial	74.27	12.33	98.47	29.70	80.60	23.41	76.33	13.78	3.469(3)	.022	b > a b > c b > d	.159
TMT_B	54.40	20.52	59.53	20.63	87.27	83.13	47.13	10.40	1.206(3)	.316	No differences	

IGD: Internet Gaming Disorder; GD: Gambling Disorder; AUD: Alcohol Use Disorder; HC: Healthy Control; IED: Intra–Extra Dimensional Set Shift; TMT_B: Trails Making Test type B.

^a^ Internet Gaming Disorder; ^b^ Gambling Disorder; ^c^ Alcohol Use Disorder; ^d^ Healthy Control

## DISCUSSION

The aim of the present study was to test the impulsivities and compulsivities of behavioral addictions, including IGD and GD, by directly comparing them with AUD andHC groups.

Impulsivity represents a multidimensional construct, which has been defined as “a predisposition toward rapid, unplanned reactions to stimuli with diminished regard to the negative consequences of these reactions” (Brewer & Potenza, 2008; Moeller et al., 2004). In the present study, we assessed the impulsivities of the participants in terms of their neurocognitive profiles and personality traits. We found that the IGD group showed a decreased proportion of successful stops on the stop-signal test compared with the HC group, which was comparable to that of the AUD patients. Furthermore, the IGD group scored significantly higher on the BIS-11 as a whole than did the HC group, which was also comparable to that of the AUD patients.

Previous studies have shown increased impulsivities in IGD patients compared with HC, as measured by the stop-signal test of the CANTAB (assessing response inhibition) and the BIS-11 (Cao et al., 2007; Choi et al., 2014; Dalbudak et al., 2013; Ding et al., 2014). Similarly, response impulsivity is elevated in individuals with substance use disorders. Longer reaction times on the stop-signal test have been found in association with cocaine (Fillmore & Rush, 2002; Li et al., 2006) and alcohol dependence (Goudriaan, Oosterlaan, de Beurs & van den Brink, 2006; Lawrence et al., 2009) and methamphetamine abuse (Monterosso et al., 2005). Alcohol-dependent patients also have been shown to commit more commission errors than controls on go/no-go tasks (Goudriaan, Oosterlaan, de Beurs & van den Brink, 2005; Kamarajan et al., 2005). Our findings are in accordance with previous studies reporting that patients with IGD and SUD have higher impulsivities compared with HC.

Interestingly, we found that the GD group scored significantly lower on the BIS-11 as a whole than did the IGD and AUD groups. In addition, although cognitive impulsivity was significantly higher in the GD group compared with the IGD group, the motor, non-planning and total impulsivity scores were significantly higher in the IGD and AUD groups than in the GD group. This implies that there are slight differences among behavioral addictions in terms of im-pulsivity (e.g., the GD group showed a greater ability to make relatively quicker decisions and to act with more contemplation and had fewer issues with future planning compared with the IGD group). Another possible explanation is that the differences in impulsivities between the GD group and the AUD or IGD groups could be due to the addictive rather than impulsive behaviors of GD, and this hypothesis is supported by some previous reports showing no differences compared with controls (Dannon, Shoenfeld, Rosenberg, Kertzman & Kotler, 2010). Although the GD group showed less overall impulsivity compared with the IGD and AUD groups in this study, it is possible that GD patients may exhibit more impulsive tendencies exclusively following domain-specific stimuli in relation to their gambling behaviors in real-world situations (Reid, Cyders, Moghaddam & Fong, 2013). Thus, stimuli-specific response inhibition should be considered for the treatment of GD, such as the management of high-risk situations.

It has been proposed that the pathologies of addictive disorders, including GD and AUD, involve a shift from impulsivity to more habit-driven compulsivity (Brewer & Potenza, 2008; Grant & Kim, 2014; Koob & Le Moal, 1997). Compulsivity has been defined as “actions inappropriate to the situation that persist and have no obvious relationship to the goal” (Leeman & Potenza, 2012). Previous research has shown that individuals with GD have greater perseveration responses and similar compulsivities as those with SUD, although findings have been more consistent involving cases of GD, and some negative findings have been reported for AUD (Goudriaan et al., 2005, 2006; Leeman & Potenza, 2012). In this study, we found that the GD group made more significant errors compared with the IGD and HC groups on the intra–extra dimensional set shift test from the CANTAB, which was designed to assess compulsivity in clinical trials. A previous study reported that pathological gambling subjects committed more perseverative errors on the Wisconsin Card Sorting Task, which is another measure of cognitive flexibility. These deficits in cognitive flexibility may cause the pathological gambling subjects to exhibit persistent damaging behaviors (Potenza, Kosten & Rounsaville, 2001). These findings regarding the compul-sivity of the GD group support previous studies indicating that perseveration may be more of an inherent aspect of GD than SUD (Grant &Kim, 2014; Leeman&Potenza, 2012).

One unexpected finding of this study was the comparable performances of the GD and HC groups on the Trail Making Test type B. The Trail Making Test measures cognitive flexibility, alternating attention, and behavioral inhibition, and previous studies have shown impaired cognitive flexibility in GD patients (Odlaug, Chamberlain, Kim, Schreiber & Grant, 2011). One possible explanation for our negative finding is that our GD subjects were young and relatively highly educated and presented with short illness durations (3 years on average). Thus, further studies are needed to confirm these findings with older patients who may have tendencies toward greater levels of cognitive inflexibility.

Another important finding in this study was that the IGD group did not differ from the other groups in terms of compulsivity. Therefore, we may postulate that the IGD group shares features of impulsivity rather than com-pulsivity with other addictive disorders (as was observed in some IGD patients), which was not observed in the GD group.

The present study has several limitations. First, the sample size was small, and thus, limited findings were attained. Second, we included only male patients, so our findings may not be generalizable to females. Lastly, all of the assessments were conducted at one time, and possible time-related changes cannot be ruled out.

However, this study has some strengths. For example, we included male patients unmedicated for their condition to control for confounding factors such as medication and gender. We also included patients who sought treatment for IGD, GD or AUD, and diagnoses were made by psychiatrists to accurately identify individuals with GD and IGD. As reflected by the PGSI, IAT and AUDIT-K scores, all groups exhibited moderate-to-severe addictive behaviors. Thus, our study has the advantage of identifying the characteristics of treatment-seeking patients with severe psychopathologies, making our findings relevant in clinical settings. We also enrolled only young patients with AUD and GD who were comparable to the IGD patients in terms of age, enabling a direct comparison among the three groups.

## CONCLUSIONS

Overall, this study suggests that individuals with IGD show higher levels of impulsivity, which are comparable to those of the AUD group. The GD group showed levels of trait impulsivity of cognitive domains similar to those of the IGD and AUD groups. In contrast, the GD group showed higher levels of compulsivity. These findings indicate that IGD shares its characteristic impulsivity with other addictive disorders. This study paves the way toward the elucidation of the similarities and differences between substance addiction disorders, such as AUD, and behavior addiction disorders, such as IGD and GD. In addition, these findings may aid the understanding of differences not only in the categorical aspects between IGD and GD but also in the impulsivity– compulsivity dimensional domains. Further, they may facilitate the targeting of specific symptoms for the more effective treatment of each behavioral addictive disorder.
